# The Dearth of Candidates for Poor-Law Posts

**Published:** 1912-12-14

**Authors:** 


### THE DEARTH OF CANDIDATES FOR POOR-LAW POSTS.

We drew attention in our Notes recently to the state of affairs which is confronting those responsible for the staffing of the Poor-Law infirmaries in this country through the wretchedly inadequate pay that is offered throughout the whole medical service, and especially to candidates for the junior posts. Exactly how the indoor Poor-Law Medical Service compares with the Public Health, Fever, and Mental Hospital Services was very clearly brought out in the figures quoted by Dr. H. W. Bruce, which enable a categorical comparison to be made. It would seem from the Report which he presented to the Southwark Board of Guardians that they are beginning to be exercised in spirit by awakening to the well-known fact that candidates for the junior resident Poor-Law posts have not had any previous experience, save the necessary modicum involved by one who has passed through the recognised curriculum that is required for registration. The truth is, of course, that experience, which for many reasons may prove useful in private practice, is the only thing which such posts have to offer to the newly qualified medical man. The chances of experience, however, are uncertain in days when improper provision for surgical work is still sometimes heard of, when, indeed, sometimes there is no operation-room at all, and when, in short, the progressive medical officer, to whose insistence on improvements the wonderful advance of the Poor-Law infirmary has been due, is met so often with misapprehension on the part of Boards of Guardians. Many still fancy that it is cheaper to glut the infirmary with chronic cases than to spend money on up-to-date improvements which ever aim at shortening the stay of patients and at hastening the day when they may be discharged as cured.

It says a great deal for the magnificent opportunities which improved conditions of service would undoubtedly bring, that, handicapped as they have been, medical officers have been found hitherto as rival competitors for election. The time has come now, however, when the improvement of the conditions of the medical service cannot be delayed any longer; for the position in which the Southwark Guardians have been placed is one which, apart from the striking criticism of existing defects that it offers, no Board of Guardians can face with either equanimity or even dignity. If only they would recognise the truth, it is the Guardians themselves who are equally victims with the medical officers whom an evil custom has led them to under-pay. A badly-paid staff is an expensive staff, and an inefficiently equipped organisation that produces a crop of chronics is an extravagance such as no Board the electors of which fully apprehended the facts would ever allow to be perpetuated in the interest of economically minded ratepayers. The question which at once presents itself to the more statesmanlike Boards of Guardians is: Where shall reform begin? We have no hesitation in saying by raising the salary of the medical officers. Indeed the logic of events, and the dearth of candidates, is exercising stronger pressure than any argument of ours in this very direction. Successful administration in the end always resolves itself into a capable and efficient staff. But capacity and efficiency, just because they breed their kind, must have a reasonable remuneration.

